# Hypoxic Roadmap of Glioblastoma—Learning about Directions and Distances in the Brain Tumor Environment

**DOI:** 10.3390/cancers12051213

**Published:** 2020-05-13

**Authors:** Agnieszka Bronisz, Elżbieta Salińska, E. Antonio Chiocca, Jakub Godlewski

**Affiliations:** 1 Department of Neurosurgery, Harvey Cushing Neuro-Oncology Laboratories, Brigham and Women’s Hospital, Harvard Medical School, Boston, MA 02115, USA; abronisz@bwh.harvard.edu (A.B.); eachiocca@bwh.harvard.edu (E.A.C.); 2 Department of Neurochemistry, Mossakowski Medical Research Centre, Polish Academy of Sciences, 02-106 Warsaw, Poland; esalinska@imdik.pan.pl

**Keywords:** glioblastoma, hypoxia, transcriptome, autophagy, immune response

## Abstract

Malignant brain tumor—glioblastoma is not only difficult to treat but also hard to study and model. One of the reasons for these is their heterogeneity, i.e., individual tumors consisting of cancer cells that are unlike each other. Such diverse cells can thrive due to the simultaneous co-evolution of anatomic niches and adaption into zones with distorted homeostasis of oxygen. It dampens cytotoxic and immune therapies as the response depends on the cellular composition and its adaptation to hypoxia. We explored what transcriptome reposition strategies are used by cells in the different areas of the tumor. We created the hypoxic map by differential expression analysis between hypoxic and cellular features using RNA sequencing data cross-referenced with the tumor’s anatomic features (Ivy Glioblastoma Atlas Project). The molecular functions of genes differentially expressed in the hypoxic regions were analyzed by a systematic review of the gene ontology analysis. To put a hypoxic niche signature into a clinical context, we associated the model with patients’ survival datasets (The Cancer Genome Atlas). The most unique class of genes in the hypoxic area of the tumor was associated with the process of autophagy. Both hypoxic and cellular anatomic features were enriched in immune response genes whose, along with autophagy cluster genes, had the power to predict glioblastoma patient survival. Our analysis revealed that transcriptome responsive to hypoxia predicted worse patients’ outcomes by driving tumor cell adaptation to metabolic stress and immune escape.

## 1. Introduction

Malignant tumors of the brain, such as glioblastomas, are among the most feared ones. They progress fast, killing about half of the patients within a year from the diagnosis [1]. Unlike for other types of cancers, survival rates for glioblastoma have not improved much in decades, even with a recently incorporated approach known as immunotherapy that instructs the patient’s immune system to recognize and kill cancer cells [2,3]. The main reason for such dismal outcomes is that glioblastomas are highly heterogeneous, which manifests at many levels: genetic, epigenetic, developmental, and microenvironmental [4]. Finding out which cell types in a heterogeneous tumor are eradicated by the therapy and which resist it, will provide the context on the obstacles that boost in vivo resistance. Yet, the preclinical models do not address these difficulties, so therapeutic strategies that work without a glitch ex vivo meet considerable barriers in the clinic [5]. 

There are many hurdles for brain tumor therapy: heterogeneity, acidic microenvironment, or macrophage clearance and immunosuppression [6,7], and all of them are, to a certain degree, linked to the hypoxic environment [8]. So, considering the fundamental role of hypoxia in intra-tumoral interactions, the identification of targets that drive adaptation to the hypoxic niche is crucial for a fuller understanding of the genesis, progression, and therapeutic resistance of glioblastoma. 

Transcriptome profiling of both: glioblastoma tissue specimens [4] and single-cell analysis, including glioblastoma stem-like cells, revealed the presence of distinguishable tumor subtypes and cellular hybrids [8,9,10]. These findings have clinical implications, as revealed transcriptional programs act in tumor anatomic site-dependent and hypoxia-dependent contexts, enhancing tumor heterogeneity and promoting therapy resistance [11,12]. 

Hypoxia also promotes tumor immune evasion via diverse mechanisms. These include cellular adaptation, autophagy, and immune-suppressive secretion [13,14,15,16,17]. The hypoxic environment cannot be eradicated, therefore the signaling response to hypoxia is exploited to therapeutic advantage [18,19,20]. Two transcription factors primarily coordinate response to hypoxia: Hypoxia-Inducible Factor 1 alpha (HIF1A) and Endothelial PAS Domain Protein 1 (EPAS1, also known as HIF2A). In glioblastoma cells, the destabilization of these proteins drives the loss of stemness, inhibits tumor growth, and favors improved outcomes [21]. Both HIF1A and EPAS1 proteins also coordinate metabolism, proliferation, differentiation, and development of immune T cells [22]. As a result of that and the lack of cancer specificity in the targeting of hypoxic response, no selective inhibitors of *HIF1A/EPAS1* are currently clinically approved.

Clearly, the adaptation of cancer cells to constantly evolving conditions of their microenvironment during tumor progression requires dynamic and flexible mechanisms. Yet, there is often insufficient understanding of the intra-tumoral evolution of such adaptation and co-operation between cancer cells that result in hypoxia- and autophagy-driven survival. To shed some light on the link between response to hypoxia and cell survival or death, we analyzed drivers and effectors of hypoxic response, also in the context of a recently rapidly developing immunotherapy strategy. 

## 2. Results

### 2.1. Hypoxic Zone—Gain and Loss of Transcriptome

**Figure 1:**
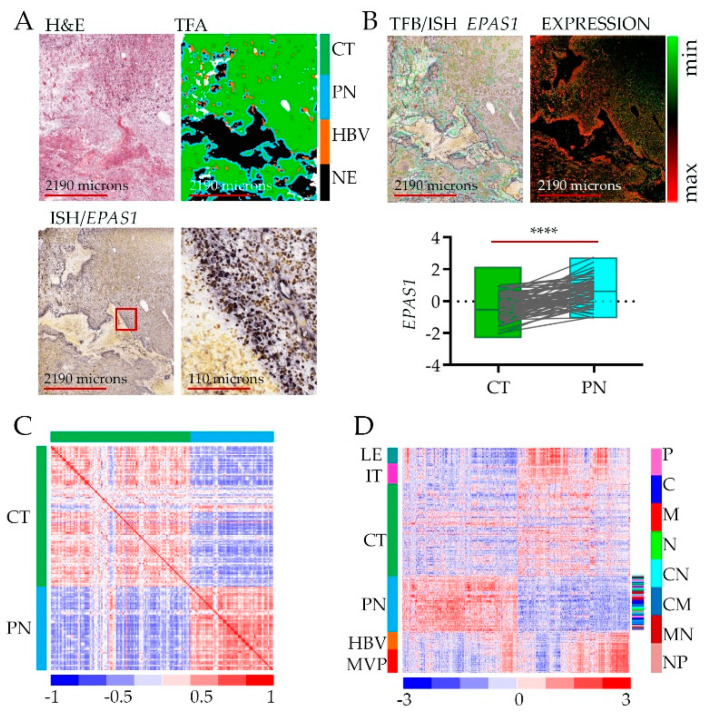
Gene expression analysis in peri-necrotic feature using Ivy Glioblastoma Atlas Project (Ivy GAP database): (**A**) Reference hematoxylin and eosin staining (H&E) section from tumor W21-1-1-B (top left), converted to tumor feature anatomy (TFA) color map (top right), labeled using in situ hybridization (ISH) probe against Endothelial PAS Domain Protein 1 (EPAS1) gene (bottom); the red square on the left panel indicates the site of magnification on the right. Color code: green—cellular tumor (CT); blue—peri-necrotic zone (PN); orange—hyperplastic blood vessels (HBV); black—necrosis (NE). (**B**) Reference H&E section from tumor W21-1-1-B merged with tumor feature boundary (TFB) of an anatomic region, labeled using ISH probe against EPAS1 gene (top left); and ISH EPAS1 signal converted to fluorescent signal (top right); normalized log fold change RNA-seq expression data of EPAS1 for CT (*n* = 111) and PN (*n* = 66) features, pairwise two-tailed *p*-value (*n* = 65) is shown. Sample pairs derived from two anatomic features from the same individual are denoted by the grey lines (**** *p*-value > 0.00001). (**C**) A correlation matrix is showing correlation coefficients between gene expression (*n* = 2707) in CT and PN zones (*r* = 0.2204). (**D**) Hieratical clustering of genes identified as significantly deregulated between CT and PZ (*n* = 2707) in all anatomic features of glioblastoma: leading edge (LE), infiltrating tumor (IT), CT, PN, HBV, and microvascular proliferation (MVP). Glioblastoma subtype classification in the PN zone is shown in color code shown to the right (proneural—P, classical—C, mesenchymal—M, neural—N).

To show how assessments between RNA-seq and in situ hybridization (ISH) were performed [23], we present representative microdissection specimens and tumor feature borders (Figure 1A). As an example, we retrieved data for the *EPAS1* gene (Figure 1A, bottom) to show the association of its expression with the hypoxic niche. Even though the level of *EPAS1* (likewise for *HIF1A*) is regulated mostly by protein stabilization [24], the transcript shows significant enrichment in the peri-necrotic, hypoxic zone (Figure 1B).

Differential gene expression analysis revealed a total of 2707 genes whose expression was enriched or depleted in the PN hypoxic niche vs. CT (Figure 1C, Table S1). The original analysis of Ivy GAP datasets revealed that samples from the same type of anatomic feature, (whether derived from the same or different tumors with diverse subtype classification) were more alike than samples from another type of anatomic feature of the same tumor [23], which indicates that intra-tumor heterogeneity surpassed inter-tumor heterogeneity. Similarly, gene signature from the PN anatomic feature distinguishes from other types of anatomic features of the same tumor regardless of tumor origin and molecular subtype classification (Figure 1D, Table S1). Thus, intra-tumoral geography of the brain tumor environment imposed by hypoxia contributes significantly to inter-tumor heterogeneity. 

Several pathways are deregulated in response to low oxygen. That often depends on cell type and the availability of nutrients. In fact, gene the most positively correlated with *EPAS1* in the hypoxic zone is *PRKAA2* encoding for alpha 2 subunit of AMP-activated protein kinase (AMPK) kinase complex—a conserved evolutionary sensor of energy availability [25,26]. Yet the same gene is the most inversely associated with *EPAS1* expression in the IT/LE feature (Figure S1A,B), suggesting that cell-dependent adaptation to hypoxia has an impact on glucose metabolism and in turn drives the adaptation of glioblastoma cells to anatomic features via a switch between proliferation and migration [27]. This premise is strengthened by the fact that the neurogenic locus notch homolog protein 3—*NOTCH 3* is the most inversely correlated gene to *EPAS1* in PN zone (Figure S1A,B) while they are both highly expressed in endothelial cells enriched in HBV/MVP zones, clearly drawing the border of vascular responses to limited oxygen. Additionally, an independent inverse correlation of *NOTCH3* and *PRKAA2* underscores the proposed negative loop between them [28,29,30]. 

This straightforward yet insightful analysis of genes associated with the hypoxic zone prompted us to perform a more detailed analysis of gene expression and their function associated with hypoxia in cancer cells.

### 2.2. Gene Ontology Analysis of the Hypoxic Zone—Adaptation or Defense

**Figure 2:**
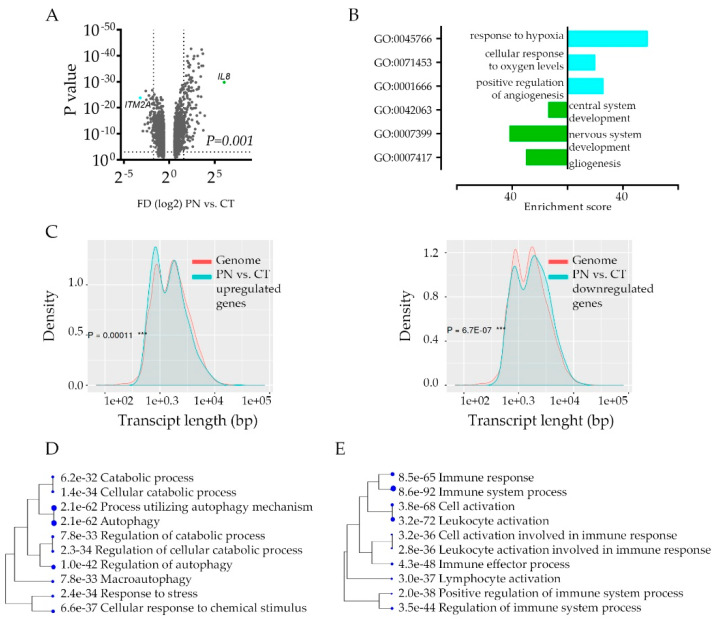
Gene Ontology enrichment analysis in hypoxic feature using Ivy GAP database: (**A**) CT vs. PN differential gene expression analysis (*n* = 2707) by volcano plot shows the most (*p*-value < 0.01. FD2, denoted by dashed lines) up- and down-regulated genes in the PN feature of glioblastoma tumor (*n* = 366, and 556, respectively). (**B**) Gene ontology analysis showing top three most enriched biologic functional categories of genes up- (blue) and down-regulated (green) in PN vs. CT glioblastoma anatomic feature. (**C**) The characteristics of genes down- (*n* = 366, left) and upregulated (*n* = 556, right) in PN in comparison with the rest of the transcripts in the human genome based on the transcript length. (**D**,**E**) A hierarchical clustering tree summarizing the correlation among significant: unique autophagy pathways genes (**D**, gene *n* = 64) and immune system pathway genes (**E**, *n* = 451) in PN in comparison to CT feature. Pathways with shared genes are clustered together. Bigger dots indicate more significant *p*-values.

The intra-tumoral hypoxic transcriptome signature enhances the inter-tumor heterogeneity; the functional consequences of such transcriptome deregulations are not clear. To gain an insight into the implications of hypoxia response, we took a closer look at transcriptome signature to pinpoint cancer cell hypoxia-specific genes’ function. Not surprisingly, the global analysis of genes that differ between CT and PN (Figure 2A) show profound downregulation of genes engaged in the development of the nervous system in PN zone, while the most upregulated PN zone genes’ signature show association with hypoxia response (Figure 2B, Table S2A). The characterization of PN-specific genes queried with the genome revealed significant downregulation of longer transcripts and upregulation of shorter ones (Figure 2C, Figure S2A) implicating stress-dependent instability and post-transcriptionally repressed transcripts [31].

If this is the case, downregulated genes may constitute non-specific deregulation of transcriptome profiles. We performed an analysis of the promoter sequences of PN vs. CT signature genes and compared them with those of the other genes in the genome in terms of transcription factor (TF) binding motifs. Both up and down-regulated signatures revealed significant enrichment of certain TF dependent genes, indicating considerable activation of transcriptional regulation of genes in the PN zone (Table S2B). It revealed a unique category of genes that carry out the hypoxic response, especially since the E2F family of TF, not HIF1A responsive genes, were the most enriched among TF binding motifs. Thus, we compared gene ontology classes between CT and PN regardless of gene enrichment scores. Even though each category included a different set of genes, only a few categories were unique for CT and PN area (Table S3, Figure S2B). This approach revealed the strong activation of autophagy as the most significant unique active biological process in the hypoxic area of the tumor (Figure 2D). Interestingly, in both CT and PN features, we identified over ten gene ontology categories, which include genes engaged in immune response (Table S3). As in each anatomic feature, a different set of immune response genes were deregulated, these data suggested that cells in these features use different modes of immune response communicating with the microenvironment that attracts or repel immune cells (Figure 2E).

### 2.3. Tumors’ Hypoxic Zones Require a Systemic Approach

**Figure 3:**
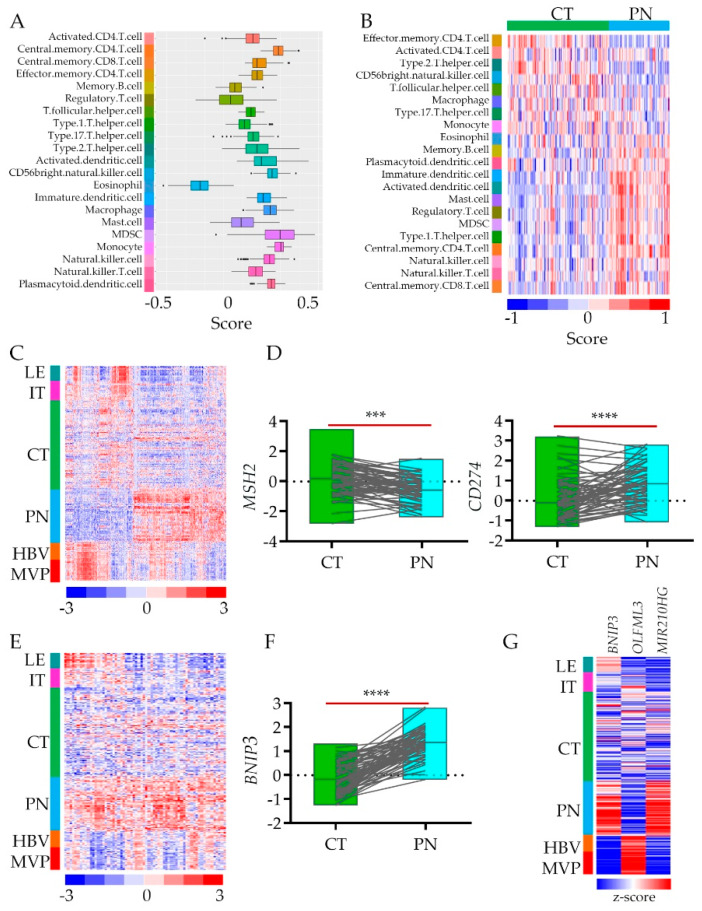
Immune and Autophagy modes enrichment analysis in hypoxic feature using Ivy GAP database: (**A**) Estimation of immune cells enrichment in PN vs. CT glioblastoma anatomic features based on a support vector machine (SVM) and the artificial immune system (AIS) algorithm using differential gene expression data (*n* = 2707). (**B**) Gene expression profiles deconvoluted into cell-type-specific sub-profiles (SVM/AIS) for analyses of relative proportions of immune cell type stratified according to their CT or PN cluster membership. (**C**) Hieratical clustering of immune mode genes identified in PN vs. CT signature (*n* = 451) in all anatomic features of glioblastoma. (**D**) RNA-seq expression data of MHS2 and CD274 genes (normalized log fold change expression data) for CT (*n* = 111) and PN (*n* = 66) features, pairwise two-tailed *p*-value between pair (*n* = 65) is shown. The grey lines denote sample pairs derived from two anatomic features from the same individual (**** *p*-value < 0.00001). (**E**) Hieratical clustering of autophagy mode genes identified in PN vs. CT signature (*n* = 64) in all anatomic features of glioblastoma. (**F**) RNA-seq expression data of BNIP3 gene (normalized log fold change expression data) for CT (*n* = 111) and PN (*n* = 66) features, pairwise two-tailed *p*-value between pair (*n* = 65) is shown. The grey lines denote sample pairs derived from two anatomic features from the same individual (*** *p*-value < 0.0001, **** *p*-value < 0.00001). (**G**) Heat map of RNA-seq expression correlation data for BNIP3, OLFML3 (*r* = −0.767), and MIR210HG (*r* = 0.734). All glioblastoma anatomic features are shown.

The term “cold tumor” refers to the one that is poorly infiltrated by the immune system’s cells. Even though glioblastoma belongs to “cold” ones, the anatomic tumor features’ gene signatures show differential expression of immunoediting genes [23]. To assess cell-type proportions between cellular and hypoxic tumors, we dispersed tissue gene expression data to estimate immune cell type enrichment in CT vs. PN tissues [32] (Table S1). Using cell-type-specific gene expression references [33], we showed that while genes associated with lymphocytes such as CD.4.T cells are present in both anatomic features, the regulatory T cells are enriched in a hypoxic zone, which may affect CD.4.T cells activity (Figure 3A,B). On the other hand, macrophages do not infiltrate the hypoxic environment while natural killer (NK) cells show up there (Figure 3A,B). Yet not only immune cells, but also immune cells attractants and repellents are differentially present in CT vs. PN (Figure 3C). As proof of concept, we selected two genes associated with these features, respectively. We retrieved data for the *MSH2* (gene encoding DNA mismatch repair protein MSH2) and *CD274* genes (encoding programmed death-ligand 1 (PD-L1) also known as cluster of differentiation 274 (CD274) or B7 homolog 1 (B7-H1)) from CT and PN anatomic features (Figure 3C) to show the association of their expression with the hypoxic niche (Figure 3D). Their inverse expression correlation in The Cancer Genome Atlas glioblastoma (TCGA GBM) bulk data (Figure S3B) suggested that low expression of immune response cell attractants and upregulation of their repellents result in an immune cold hypoxic environment.

These observations may thus explain why approaches based on instructing immune cells on how to kill tumor cells that are useful in immune-activating cancers, have not led to significant breakthroughs in brain tumor clinical trials. Such a signature does not explain why other immune approaches, e.g., oncolytic viruses, which reprogram cancer cells to attracted immune cells, have not led to long-term survival either. Thus we included in the analysis the process of autophagy prevalent in hypoxia that imposes the clearance of protein aggregates but also viral particles [34].

The genes associated with the process of autophagy (Figure 2D) are enriched in the hypoxic areas of the tumor. Although some autophagy genes are also expressed in LE, IT, and HBV, this pathway is shut down in the CT areas of glioblastoma (Figure 3E). As autophagy can drive both: cellular apoptosis and survival, it raises the question of what their role is in the hypoxic environment of glioblastoma tumors. Is this process turned on in response to stress as a defense strategy or adaptation? Even though what we observed is just a snapshot picture of the transcriptome, the possibility that autophagy destroys glioblastoma cell is unlikely, as the autophagy promotes apoptotic cell death while the death of glioblastoma cell is usually a necrotic one [35]. While the reactivation of programmed cell death represents a promising therapeutic strategy [36], the apoptotic pathways’ gene signature was not prevalent in the PN zone. As a proof of concept, we selected the most upregulated autophagy responsive gene: *BNIP3* (encoded BCL2 Interacting Protein 3) (Figure 3F). We queried its expression with genes expressed differentially between CT and PN.

Interestingly, the gene most positively correlated with *BNIP3* was *MiR-210HG* (*r* = 0.734) – (non-coding RNA MIR210 host gene) hypoxia adaptative response gene [37]. On the contrary, encoding olfactomedin-like protein 3 (*OLFML3*) is a gene the most inversely associated with *BNIP3* expression (*r* = −0.767) (Figure 3G, Figure S3C). As *OLFML3* is endothelial and microglia cell receptor [38], lack of its expression in PN underlined poorly developed vasculature and lack of immune cell infiltration of hypoxic tumor zone with autophagy program turned on, as the recruitment of immune-suppressive microglia into CT zone constitute a formidable barrier for lymphocytes thereby preventing their infiltration into the zone.

### 2.4. The Impact of Hypoxia on the Outcome of Glioblastoma Patients

**Figure 4:**
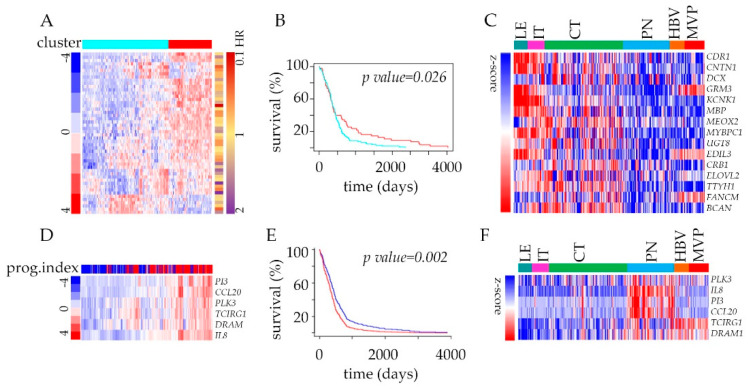
Survival estimator analysis of glioblastoma patients from TCGA based on the cluster membership of genes in the Ivy GAP dataset: (**A**) Heatmap with color annotations according to profile similarity (blue/red) of 50 most down-regulated genes (based on differential analysis of CT and PN zones, *n* = 2707) annotated with Hazard Ratios (HR red-violet) from Cox analysis. (**B**) Survival analysis of TCGA GBM samples dataset stratified according to their cluster membership (blue/red) from panel (**A**) using Kaplan–Meier analysis. (**C**) Heat map of RNA-seq expression correlation data for 15 (with *p*-value < 0.01) from 50 gene signature in all glioblastoma anatomic features is shown. (**D**) Hierarchal clustering analysis of a full cohort of TCGA GBM samples dataset stratified by inspecting the status of genes (*n* = 6) with the most prognostic index (based on autophagy (*n* = 64) and immune mode (*n* = 451) genes’ inputs). (**E**) Survival analysis based on the impact of the 6-gene from panel (**D**) with prognostic index hazard ratio = 1.61 and log-rank *p*-value = 0.002 in a full cohort in TCGA GBM samples dataset using Kaplan–Meier analysis. (**F**) Heat map of RNA-seq expression correlation data for six genes from panel (**D**) signatures in all glioblastoma anatomic features is shown.

The analysis of transcriptome has a long history of clinical implications, and in addition to discovering new biomarkers or therapeutic targets, it can identify prognostic markers as well. To funnel our analysis into the context of clinically relevant targets, we developed an admixture model using a 2707 gene signature (Table S1) queried with TCGA GBM survival data. Genes deregulated between CT, and PN features were divided into two groups (upregulated and down-regulated) and filtered to keep the most varied molecules amongst the list of genes annotated with hazard ratios from Cox analysis. This strategy revealed significant power of genes down-regulated in PN to predict the outcome using Kaplan–Meier estimator survival analysis (Figure 4A,B, Figure S4A–E). Within the most significantly depleted genes, many are associated with cell adhesion (Figure 4C). The analysis of upregulated and down-regulated genes together did not improve the predictive power of the patients’ outcomes (Figure S4B–F). Once we narrow our analysis to genes associated with immune and autophagy modes (Table S3), the analysis of the prognostic index of these genes displayed strong predictive value. The significant improvement of patient survival was associated with the below-median expression of the selected 6-gene signature (Figure 4D,E). These include hypoxia-induced Polo-like kinase 3 (*PLK3*) engaged in the regulation of chaperone-mediated autophagy [39] and pro-inflammatory chemokine IL-8 that promotes tumor immune escape [40] (Figure 4F).

Obviously, the targeting of oxygen levels is not a feasible option, but specific pathways deregulated in cancer cells in the hypoxic environment are worth a closer look. As multiple clinical trials use immunotherapy strategies for solid tumors, where hypoxia is a hallmark of the altered metabolism, perhaps the therapeutic response will benefit from the preclinical tests with co-targeting of hypoxic adaptation mechanisms such as enhanced autophagy and rearranged immune response.

## 3. Discussion

Hypoxia promotes tumor cell survival via diverse mechanisms. Here we show that these mechanisms primarily include cellular adaptation via autophagy and immune response remodeling [14,15,16,17]. The hypoxic environment cannot be eradicated, so the signaling response to hypoxia is being used to therapeutic advantage [18,19,20]. The response to hypoxia is a primordial and conserved pathway, and so are its effectors. So even though inhibition of hypoxia-responsive EPAS1 results in the loss of stemness, inhibition of tumor growth, and favorable outcome [21], such strategy is not cancer cell-specific [22].

The role of autophagy in the survival of cancer cells has been debated extensively as some researchers view autophagy as apoptotic cell death triggering cue, while others were pointing toward its survival-promoting role during response and adaptation to microenvironmental and therapy-inflicted stressors. Our comprehensive review of data suggests the latter mode being prevalent in hypoxic regions of glioblastoma. The subpopulation of cancer cells that emerge, evolve, and thrive within hypoxic, peri-necrotic zones of these tumors seems to set in motion autophagy-related signaling as the stress-coping mechanism involved in their metabolic maintenance and proliferation [41].

To discover cell type-specific targets, we and others took a closer look at non-protein coding transcripts as candidates. Non-coding RNA (ncRNA) transcripts are not only the most prevalent in the transcriptome, but they are often expressed in a cell- and tissue-specific manner [42]. As a result of that, ncRNAs are excellent candidates for selective targeting. However, most of the annotated ncRNAs have unknown functions, thus to utilize them as the tool, the basic research has begun addressing their cell-specific expression regulation and function. Our studies provided a long-non-coding RNA candidate for the targeted attenuation of hypoxic response—HIF1A-AS2 [43]. HIF1A-AS2 is expressed in response to low oxygen in glioblastoma cells but only in those whose transcriptome is associated with the PN feature of the tumor. The HIF1A-AS2-associated transcriptome is upregulated within the hypoxic niche lacked immune cell markers [43], indicating that cells expressing HIF1A-AS2 are in immune escape mode. Yet, the comprehensive transcriptome mapping of glioblastoma does not cover most species of non-coding RNA, underscoring an urgent need to fill this gap that would yield in the discovery of numerous niche-specific targets.

Some experimental evidence suggests that tumor cells may cope with hypoxia by turning on the migratory phenotype to escape from metabolically stressful events/locations [44]. As an example of such action by glioblastoma cells, the efforts by our group provided the evidence of glucose availability-regulated feedback loop that promotes either proliferative or migratory mode, depending on the microenvironmental context. Here we argue that the adaptation to stress and the remodeling of immune surveillance drive the survival and proliferation of glioblastoma cells in hypoxic zones and that such strategy is equally, if not more important as the behavioral transition [45,46]. 

Decades of research on hypoxia in the brain provided ample examples of its participation in the neurodegeneration, including an inverse correlation between the likelihood of cancer and neurodegenerative diseases [47], which, however, did not fully explain the difference between the sensitivity and adaptation to low oxygen in non-malignant glial and neural cell and glioblastoma cancer cells, respectively. Both neural cells and cancer cells depend on the communication with cells from the microenvironment, including immune cells [48]. These also determine the response to immunotherapies based on instructing the immune cells on how to kill a tumor [2]. A considerable obstacle contributing toward the resistance to immunotherapy is enhanced immune evasion in the hypoxic tumor environment [49,50]. It became evident that the successful treatment of glioblastoma requires closer inspection of hypoxic regions within a tumor that constitutes an immunosuppressive environment. 

The buildup of hypoxia is one of the hallmarks of solid tumors, including glioblastoma. The hypoxic environment that cannot be eradicated from the brain tumor environment is undoubtedly the source of intra-tumoral heterogeneity and strong selective force that leads to the emergence of exceptionally durable and resistant tumor cell subpopulations. Thus, the cell’s capability to respond and adapt to hypoxia is an essential pro-survival trait, whose nuts and bolts require a better understanding for the development of targeted therapies, including immunotherapy. Our metadata analysis-based findings identifying the selective rise of the autophagy within the region, along with the remodeled immune response, shed new light on the adaptative strengths of cancer cells withstanding the hypoxic environment. These transcriptome rearrangements point toward the repositioning of metabolism and immune evasion as primary mechanisms promoting cancer cell survival and therapy resistance due to hypoxic signaling.

## 4. Materials and Methods

### Databases and Data Selection

The median survival for a patient with glioblastoma is 12 to 15 months, and not much improvement have been achieved for decades [1]. These tumors evolve quickly, resulting in intra-tumoral heterogeneity at both genetic and microenvironment levels and, in consequence, developing drug resistance. Even recently adopted immunotherapeutic approaches that were proven effective in other cancers, have not panned out for glioblastoma in the clinic yet. On the clinical research side, the large-scale efforts resulted in the cataloging of multi-omics datasets. Designed to depict the genomic alterations in human glioblastoma [4], they revealed astonishing variability and heterogeneity of glioblastoma cell transcriptome [8] and tumor geography [23]. These last strategies equipped us with a navigation tool, which we will operate to explore the hypoxic niche of glioblastoma.

To create the hypoxic map, we first performed differential expression analysis using the data from Ivy Glioblastoma Atlas Project (Ivy GAP). This clinical and genomic database was created using 41 glioblastoma grade IV patient cohort and 122 RNA samples (see Table S1) that were Laser Micro-Dissected (LMD) into five structural groups using in situ hybridization (ISH) panels to identify regionally enriched sets of genes sets. In brief, we used data plotted in the tumor’s anatomic features (based on ISH), followed by transcriptome analysis of LMD material and RNA sequencing (RNA-seq). As the copy number and mutation analyses indicated that most of Leading Edge (LE) and some of Infiltrating Tumor (IT), and Hyperplastic Blood Vessels and Microvascular Proliferation (HBV/MVP) samples contain non-neoplastic cells, the most valuable for our analysis was a comparison between cellular tumor (CT) and Peri-Necrotic (PN) zone (including pseudo palisading cells around necrosis). Thus, LE and HBV/MVP data are included as a reference only. Next, we selected genes’ signature using cut-off based on a significant difference in the expression of genes from these regions (false discovery rate < 0.01, *p* < 0.1, Benjamini–Hochberg corrected).

To catalog the molecular function of genes differentially expressed in the hypoxic region of the tumor, we conducted a systematic review using the Gene Ontology database created in December 2019. This strategy identified genes’ sets whose expression is enriched or depleted in the hypoxic anatomic features and described a molecular/cellular function of such signatures. Genes were eligible for inclusion if the standard nomenclature identified them as human genes in the HUGO Gene Nomenclature Committee database. To put a hypoxic niche signature into a broader clinical context, we queried the list of genes with The Cancer Genome Atlas glioblastoma (TCGA GBM) data portal, as previously described [43]. Briefly, the clinical and gene expression datasets from grade IV glioblastoma samples (*n* = 528) and control specimens (*n* = 10) analyzed by Affymetrix HT_HG-U133A microarray gene chips were queried with selected gene sets from Ivy GAP. These data allowed us to select genes with the utmost predictive outcome using Kaplan–Meier estimator survival analysis. In the Ivy GAP dataset, only three tumors from 41-patient cohort harbored Arg132 → His mutation in isocitrate dehydrogenase 1 (IDH1); thus, there was insufficient statistical power for the analysis of this mutation by anatomic feature. Consequently, we excluded patients with this mutation from the survival analysis. 

Ethical Statement: The collection of the data from TCGA GBM and the Ivy GAP was compliant with all applicable laws, regulations, and policies for the protection of human subjects.

## 5. Conclusions

Over the past decades, transcriptome-wide studies on human malignancies have produced vast amounts of data. Yet understandable need to prioritize, along with bandwidth limitations, led to inadequate depth of analysis of these valuable resources. Thus, our goal in this study was to use the metadata toolbox to gain more insight into our area of interest, i.e., transcriptomic fundaments of adaptative capabilities possessed by brain tumor cells to cope with hallmark exposure to hypoxic stress. We analyzed various layers of acquired data to identify pathways and functional signaling modes prevalent in hypoxic areas of the tumor. We identified the “autophagy” as the most unique, and the “immune response” as diversely de-regulated classes of genes prevalent in hypoxic tumors. These findings have clinical implications, as they revealed transcriptional programs acting in tumor anatomic site- and hypoxia-dependent contexts perpetuated by tumor evolution and stress buildup enhancing tumor heterogeneity and promoting therapy resistance; thus, co-targeting of hypoxic adaptations would be beneficial for glioblastoma patients.

## Supplementary Materials

The following are available online at https://www.mdpi.com/2072-6694/12/5/1213/s1, supplementary Materials and Methods and Database Information, Figure S1: Supplementary to Figure 1, Figure S2: Supplementary to Figure 2, Figure S3: Supplementary to Figure 3, Figure S4: Supplementary to Figure 4, Table S1: List of genes identified as significantly deregulated between CT and PZ in these features of glioblastoma, Table S2: (**A**) Gene ontology enrichment analysis based on hypergeometric distribution followed by FDR correction, showing the top three most enriched biologic functional categories of genes up- or downregulated in PN vs. CT. (**B**) The promoter sequences of the genes up-regulated and down-regulated compared with the other genes in the genome in terms of transcription factor (TF) binding motifs within 300 bp upstream of transcription start. Table S3: Summary of genes deregulated in PN vs. CT, grouped by functional categories defined by high-level GO terms.
