# Supplementary material for: Hypoxic Roadmap of Glioblastoma—Learning about Directions and Distances in the Brain Tumor Environment
Source: Cancers (Basel). 2020 May 13;12(5):1213. doi: 10.3390/cancers12051213 (PMC7281616; doi:10.3390/cancers12051213)
Supplement: Supplementary file 1 [file cancers-12-01213-s001.zip › cancers-790057 supplementary/Supplementary Data.docx]

*SUPPLEMENTARY DATA:*

***Supplementary Materials and Methods and Database Information:***

The software used in the study: For all studies, we used freely accessible online data resources that served as a platform for our investigation of hypoxia driving pathogenesis and treatment in glioblastoma.

TCGA (1): <https://www.cancer.gov/>

Ivy GAP (2): <http://glioblastoma.alleninstitute.org/rnaseq>

GBM biodiscovery portal (3): <https://gbm-biodp.nci.nih.gov>

dchip with R package: <http://www.dchip.org/>

Gene Ontology database: <http://www.geneontology.org/>

GlioVis (4): <http://gliovis.bioinfo.cnio.es/?ref=labworm>

HGNC database <http://www.genenames.org>

ShinyGO v0.61 <http://bioinformatics.sdstate.edu/go/>

**Details of Data analysis:** The collection of the data from IVY GAP and TCGA GBM was compliant with all applicable laws, regulations, and policies for the protection of human subjects, and necessary ethical approvals were obtained. For analysis of gene expression in glioblastoma, we used normalization of data and aggregation at the feature level as designated by the TCGA GBM the "Level3"**.** Data were analyzed using free available portals as a resource for accessing and displaying interactive views of Ivy GAP and TCGA data associated with glioblastoma (3,4).

**RNA-Seq Data Normalization:** Gene expression values were summarized as transcripts per million (TPM) and fragments per kilobase per million (FPKM). To improve the display of the website heatmap, the FPKM data matrix was further adjusted for the total transcript count using TbT normalization (5), which scales each sample based on the summed expression of all genes that are not differentially expressed. FPKM values were TbT (from a trimmed mean of M values (TMM) - TMM-baySeq-TMM) normalized in linear space, with the differential expression vector defined as TRUE if a sample was from cellular tumor and FALSE if otherwise. Sample data were then scaled in a way that the total log2(FPKM) across the entire data set remained unchanged after normalization. The result of this step was that expression levels for all genes in a particular sample were multiplied by a scalar value close to 1 (in most cases between 0.7-1.3).

**Displaying a summary of experimental data associated with selected genes:** The samples (columns on the heatmap) were annotated in two ways: first, according to the cluster membership (the optimal number of clusters was determined using NbClust); second, by inspecting the status of a prognostic index (which was computed by weight averaging the gene expressions with the regression coefficients of a multi-gene Cox proportional hazards model). The gene names were annotated with their respective Hazard Ratios in a multi-gene Cox proportional hazards model. When search results involved more than 50 genes, we filtered them by keeping the 50 genes whose expression is the most varied among the samples.

**Performing gene survival analysis:** The Kaplan-Meier survival curve analysis compared samples stratified according to gene expression levels. The default options stratified samples into two groups: those with expression levels below the median over the subgroup, and those with expression levels above the median.
For gene searches that resulted in multiple hits, we analyzed how the expression profiles impacted the survival. We performed two types of survival analyses: first, the optimum clusters were selected by the stratification of the samples according to the heatmap cluster membership (see the first annotation bar), where the optimal number of clusters is picked out algorithmically using silhouette width index. Next, we used a Kaplan-Meier model to analyze the differences in survival between groups using a log-rank statistic. Hierarchal clustering analysis of a full cohort of TCGA GBM samples dataset stratified by inspecting the status of genes with the most varied prognostic index was computed by weight averaging the gene expressions with the regression coefficients of a multi-gene Cox proportional hazards model. These analyses were performed using the "NbClust" package in R.

**Displaying heatmap clustering of gene expression data correlation:** For selected multiple hits of genes, we presented a heatmap of the correlation between the expression of genes in anatomic features. Each cell of the heatmap represented how the expression of the gene in the row, and the gene in that column are correlated, and it was annotated with the correlation value. The results were displayed as a heat map using hierarchical clustering analysis using the average linkage distance metric.

**Prognosis prediction using SVM-based methods:** SVM data classification and function approximation introduced by Vapnik (6) is a binary classifier (trained on a set of labeled patterns called training samples) (7). This SMV strategy was used to investigate the possibility of identifying different prognostic subsets of patients based on their clinicopathologic features and immunomarkers (8). We used this approach to classify the immune cell enrichment between CT and PN gene signatures.

**Quantification and Statistical Analysis**: Graphs and plots were generated, and statistical analyses were performed using GraphPad Prism 7. Statistical parameters, including the value of n, statistical test, and statistical significance (p-value), are reported in the figures and their legends. Statistical tests were selected based on the desired comparison. Unpaired two-tailed t-tests were used to assess significance when comparing data between two variances. One-way ANOVA was used to determine significance when comparing data between ≥ 3 variances; significant ANOVA results were followed by post hoc testing comparing every mean with every other mean (Tukey's multiple-comparison test). For the differential expression of global measurements (platforms), the DESeq2 software-generated adjusted p values using the Wald test with the Benjamini-Hochberg procedure to correct for multiple hypothesis testing. The Mann-Whitney test was used to compare cumulative distributions of gene fold changes between two gene sets. The characteristics of genes deregulated in PN vs. CT in comparison with the rest of the genes in the genome were analyzed by Chi-squared and Student's t-test to see if selected genes are different.

**Supplementary Figures and Figure’s Legends:**


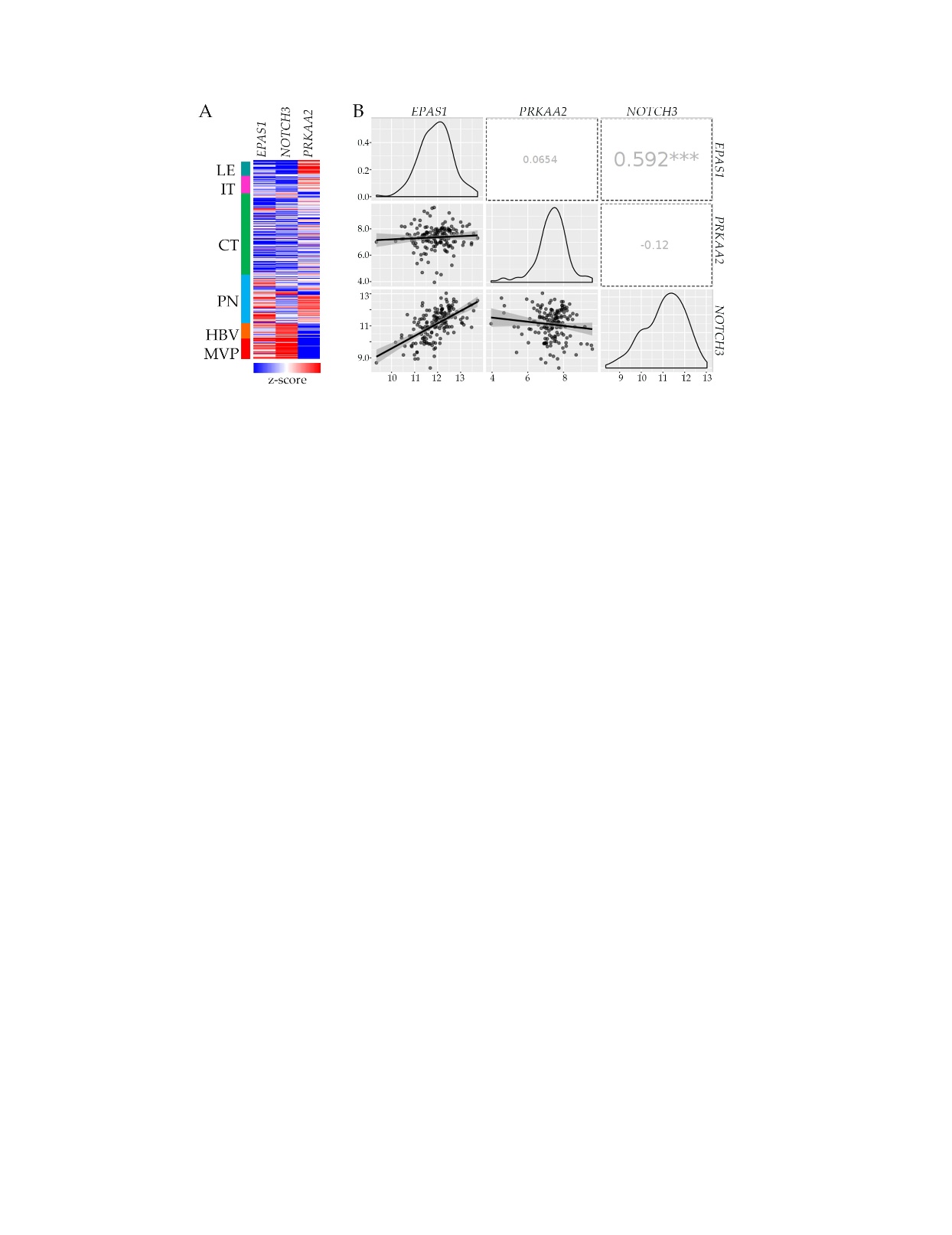


**Figure S1. Supplementary Figure to Figure 1. (a)** Heat map of RNA-seq expression correlation data for *EPAS1*, *NOTCH3,* and *PRKAA2* in all glioblastoma anatomic features.**(b)** The density plot of expression and association-correlation data for *EPAS1*, *NOTCH3,* and *PRKAA2* in glioblastoma bulk data from TCGA GBM. Pearson's product-moment correlation value and ***p-value (<0.001) is indicated.


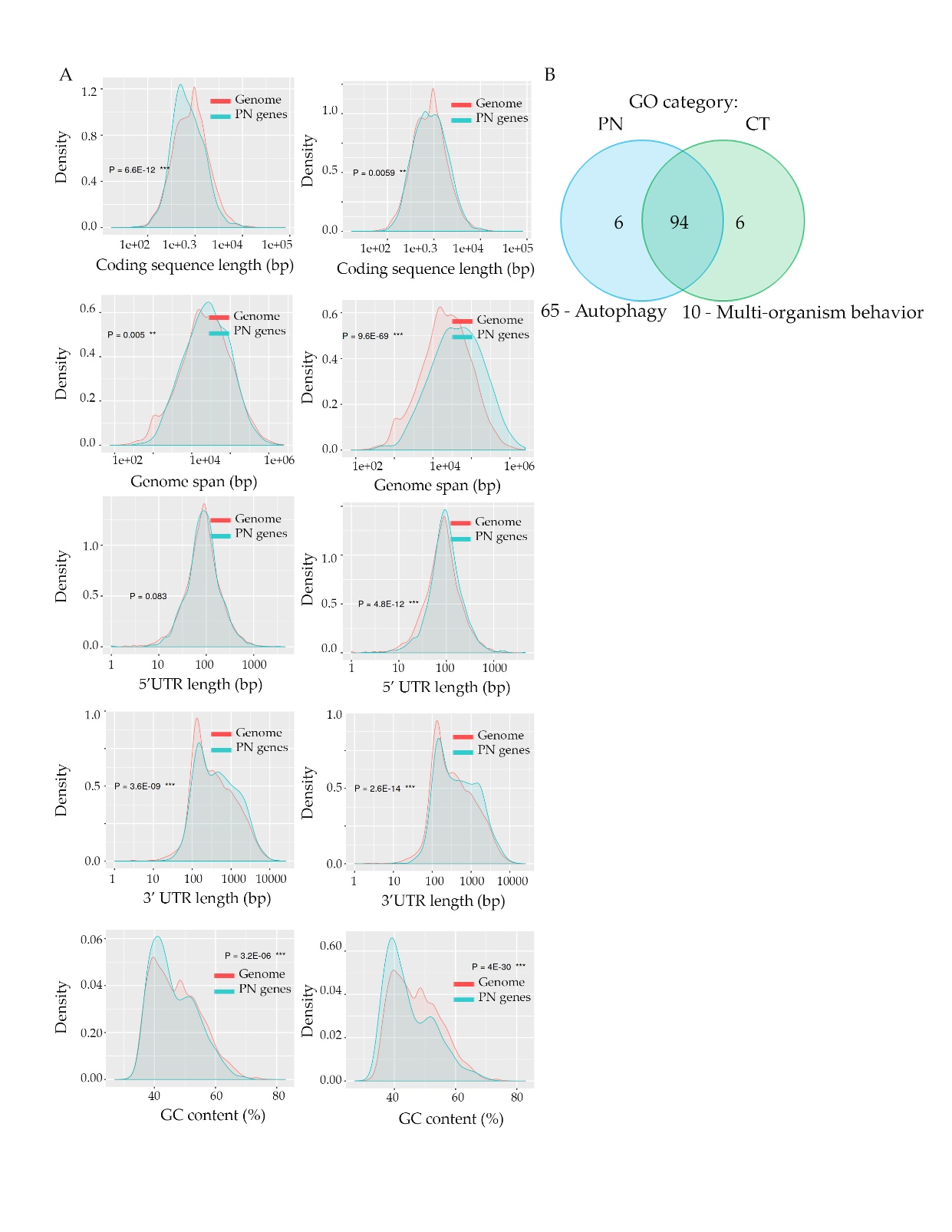


**Figure S2. Supplementary Figure to Figure 2. (a)** The characteristics of genes down- and up-regulated (n=366) in PN (n=, 556) (p value<0.01, FD>2) in comparison with the rest of the genes in the genome based on the length (in bp) of a coding sequence, genome span content, 3' and 5' UTR or percentage of GC content (respectively). **(b)** Gene Venn analysis of gene ontology category in PN and CT features, see also Supplemental Table S4.


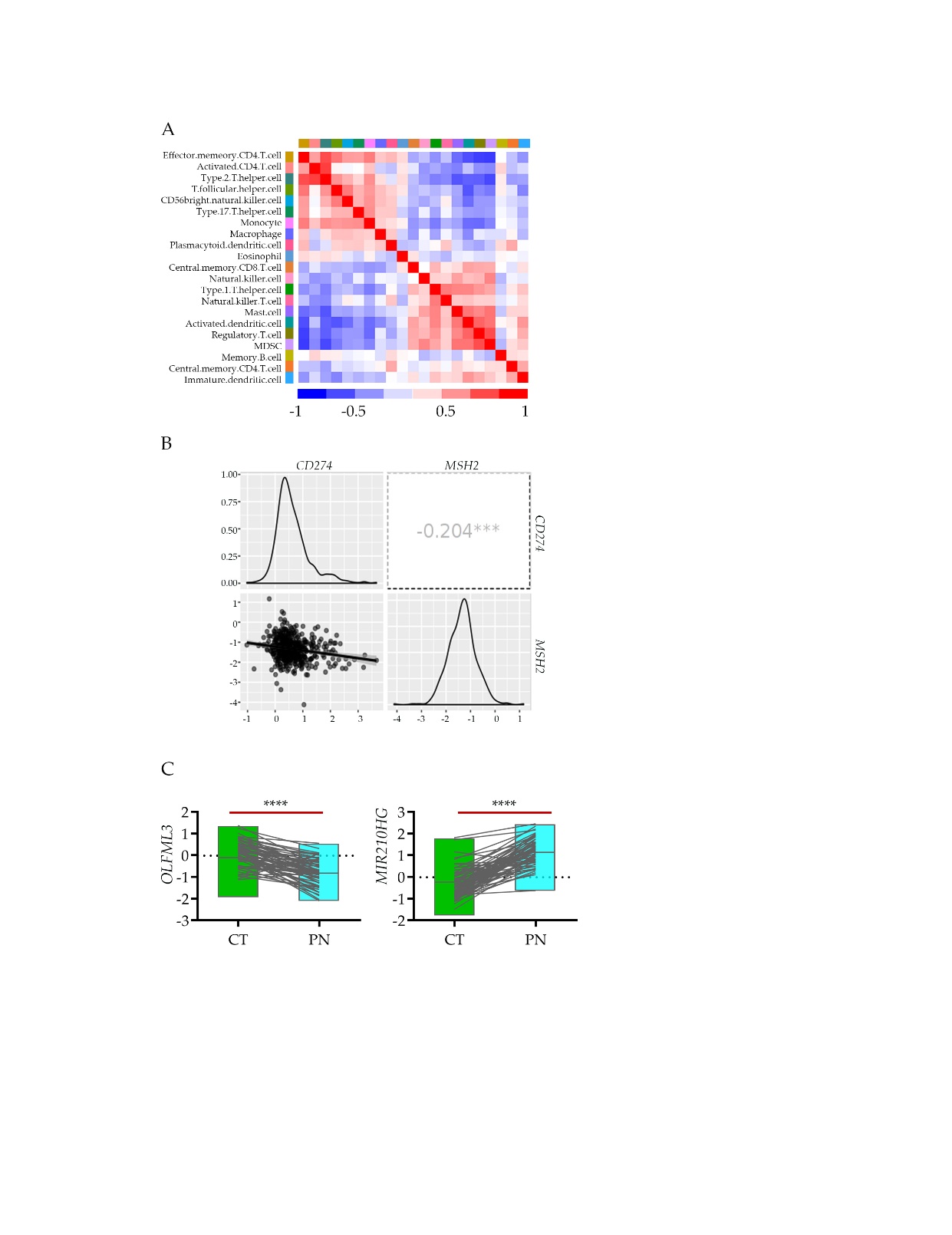


**Figure S3. Supplementary Figure to Figure 3. (a)** A correlation matrix is showing correlation coefficients between gene expression associated with cell-type-specific sub-profiles in CT and PN zones. **(b)** The density plot of expression and association-correlation between paired samples data for *CD274* and *MSH2* in glioblastoma bulk data from TCGA GBM. Pearson's product-moment correlation value and *p-value (<0.05) are indicated. **(c)** RNA-seq expression data of *OLMFL3* and *MIR210HG* genes (normalized Log fold-change expression data) for CT (n=111) and PN (n=66); pairwise two-tailed p-value between pair (n=65) is shown. The grey lines denote sample pairs derived from two anatomic features from the same individual.


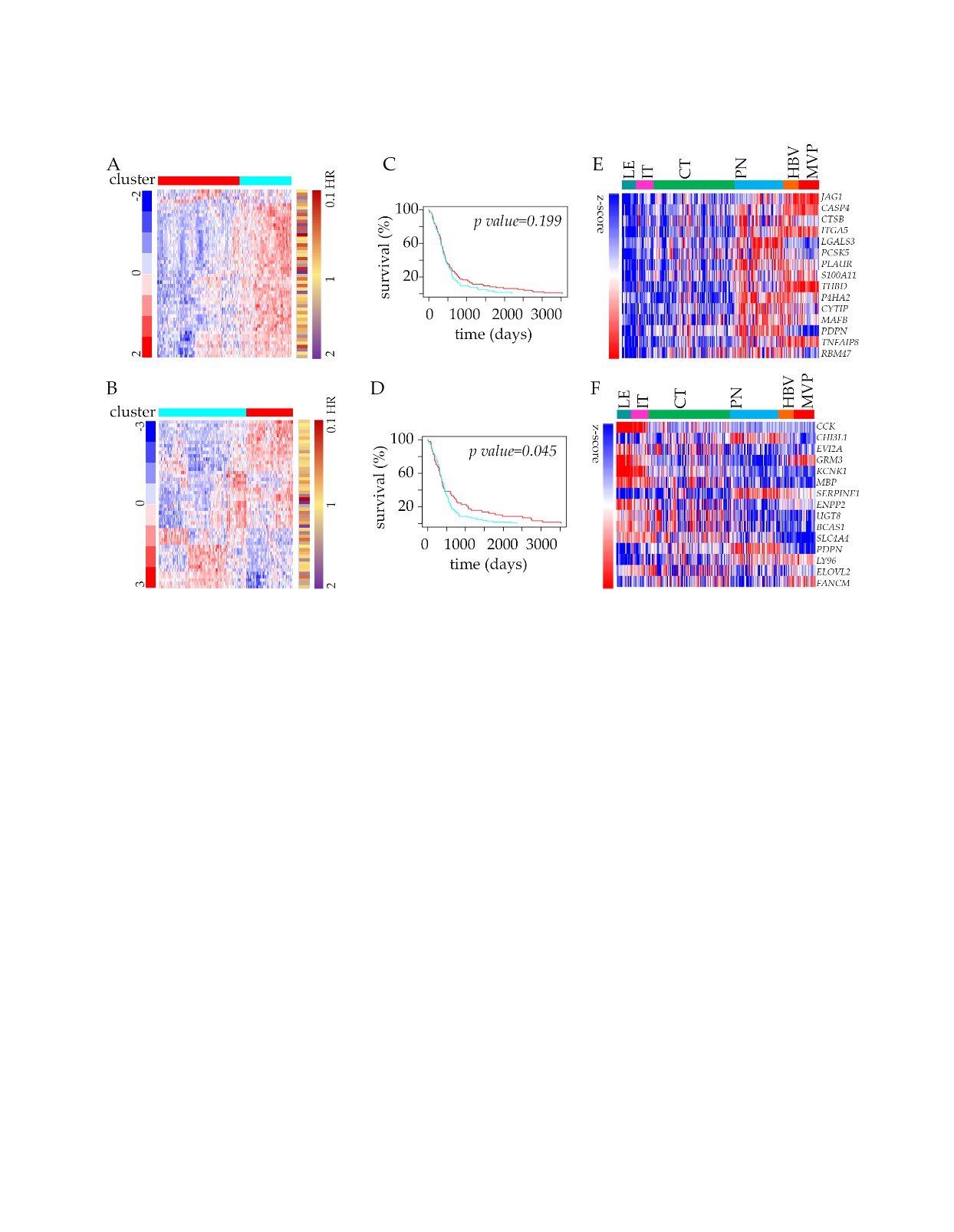


**Figure S4. Supplementary Figure to Figure 4**. **(a, b)** Heatmaps with color annotations according to profile similarity (blue/red) of 50 most up-regulated only (**a**) or both up- and down-regulated genes together (**b**) (based on differential analysis of CT and PN zone, n=2707) annotated with Hazard Ratios (HR red-violet) from Cox's based on TCGA GBM samples dataset. **(c, d)** Survival analysis of TCGA GBM samples dataset stratified according to their cluster membership (blue/red) from panels (**a**) and (**b**) using the Kaplan-Meier analysis. **(e, f)** Heatmaps of RNA-seq expression correlation data for 15 (with p value<0.01) from 50 gene signature from panels (**a**) and (**b**) in all glioblastoma anatomic features isolated by LMD.

***Supplementary Table Legend:***

**Table S1.** List of genes identified as significantly deregulated between CT and PZ (n=2707) in these features of glioblastoma.

**Table S2. A:** Gene ontology enrichment analysis based on hypergeometric distribution followed by FDR correction, showing the top three most enriched biologic functional categories of genes up- or downregulated in PN vs. CT (n= 556 and 366, respectively). **B:** The promoter sequences of the genes up-regulated (n=556) and down-regulated (n=366) compared with the other genes in the genome in terms of transcription factor (TF) binding motifs within 300 bp upstream of transcription start. An asterisk in "query gene" indicates a transcription factor coded by a gene included in the list.

**Table S3.** Summary of genes deregulated in PN vs. CT (n= 2707), grouped by functional categories defined by high-level GO terms.

***Supplementary References:***

1. Verhaak RG, Hoadley KA, Purdom E, Wang V, Qi Y, Wilkerson MD*, et al.* Integrated genomic analysis identifies clinically relevant subtypes of glioblastoma characterized by abnormalities in PDGFRA, IDH1, EGFR, and NF1. Cancer Cell **2010**;17:98-110

2. Puchalski RB, Shah N, Miller J, Dalley R, Nomura SR, Yoon JG*, et al.* An anatomic transcriptional atlas of human glioblastoma. Science **2018**;360:660-3

3. Celiku O, Johnson S, Zhao S, Camphausen K, Shankavaram U. Visualizing molecular profiles of glioblastoma with GBM-BioDP. PLoS One **2014**;9:e101239

4. Bowman RL, Wang Q, Carro A, Verhaak RG, Squatrito M. GlioVis data portal for visualization and analysis of brain tumor expression datasets. Neuro Oncol **2017**;19:139-41

5. Kadota K, Nishiyama T, Shimizu K. A normalization strategy for comparing tag count data. Algorithms Mol Biol **2012**;7:5

6. Vapnik VN. An overview of statistical learning theory. IEEE Trans Neural Netw **1999**;10:988-99

7. Wang HY, Sun BY, Zhu ZH, Chang ET, To KF, Hwang JS*, et al.* Eight-signature classifier for prediction of nasopharyngeal [corrected] carcinoma survival. J Clin Oncol **2011**;29:4516-25

8. Jiang Y, Xie J, Han Z, Liu W, Xi S, Huang L*, et al.* Immunomarker Support Vector Machine Classifier for Prediction of Gastric Cancer Survival and Adjuvant Chemotherapeutic Benefit. Clin Cancer Res **2018**;24:5574-84
