# Supplementary figures and images for: Hypoxic Roadmap of Glioblastoma—Learning about Directions and Distances in the Brain Tumor Environment
Source: Cancers (Basel). 2020 May 13;12(5):1213. doi: 10.3390/cancers12051213 (PMC7281616; doi:10.3390/cancers12051213)

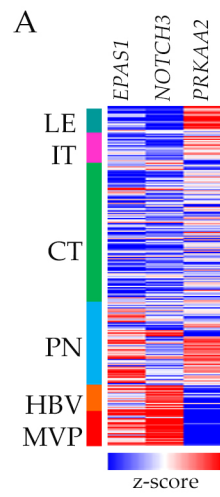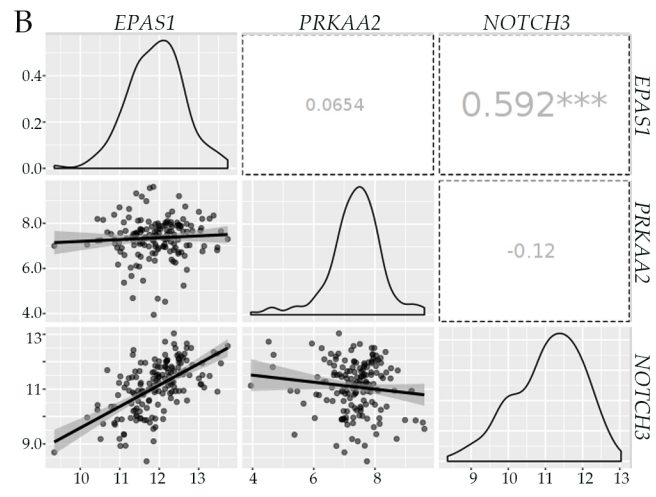

Supplement: Supplementary file 1 [file cancers-12-01213-s001.zip › cancers-790057 supplementary/Figure S1.pdf]

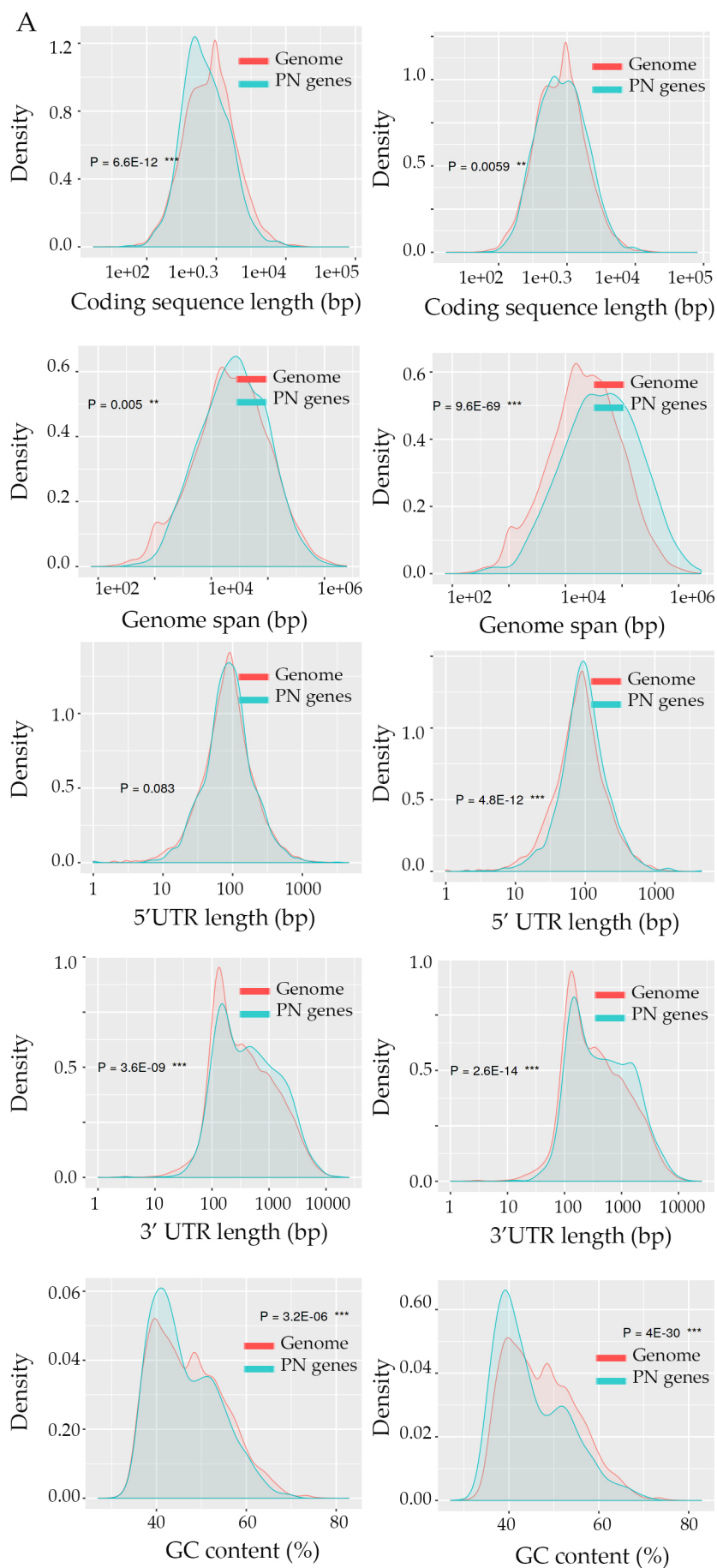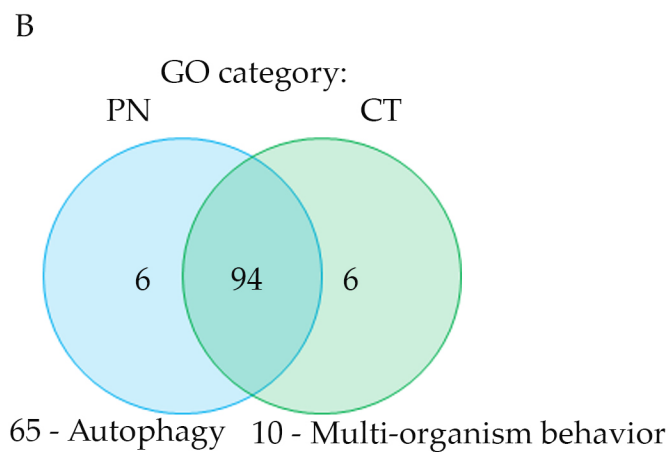

Supplement: Supplementary file 1 [file cancers-12-01213-s001.zip › cancers-790057 supplementary/Figure S2.pdf]

A

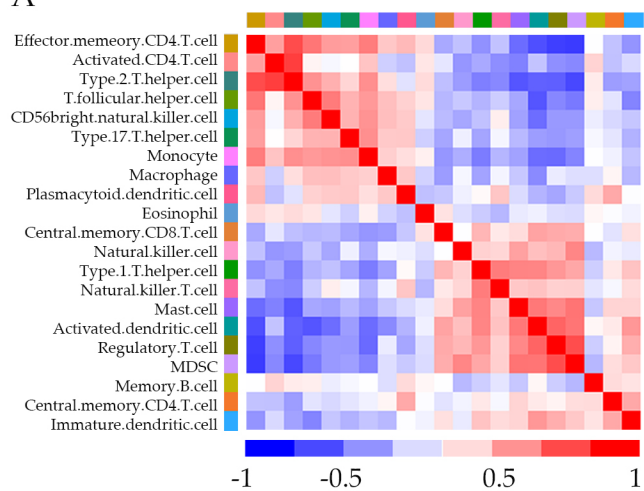

B

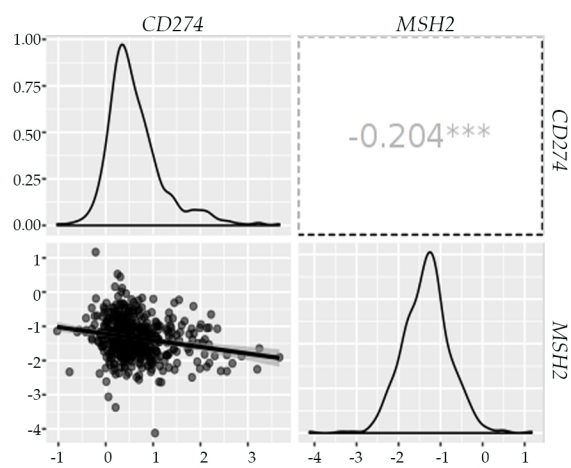

C

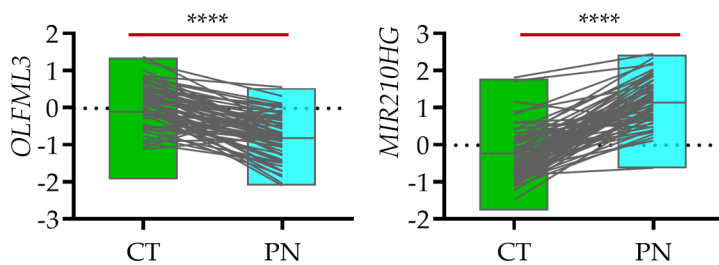

Supplement: Supplementary file 1 [file cancers-12-01213-s001.zip › cancers-790057 supplementary/Figure S3.pdf]

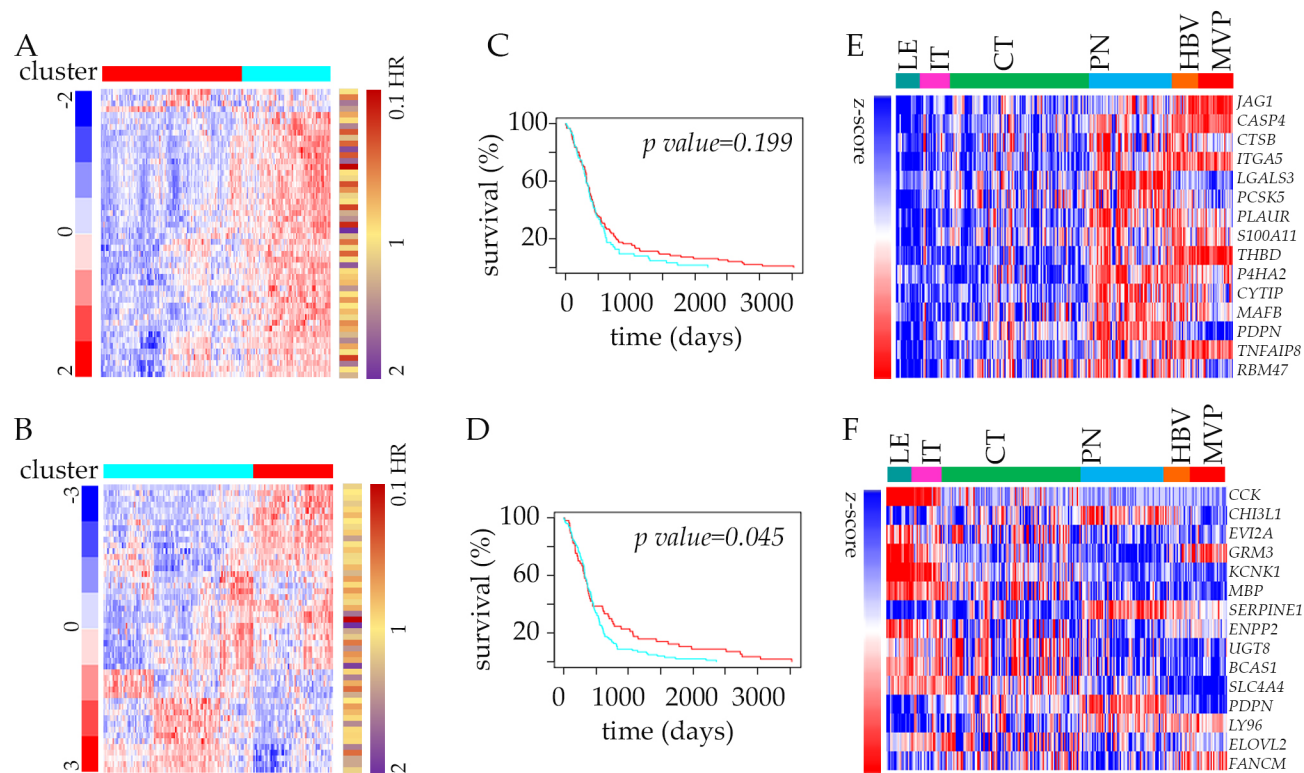

Supplement: Supplementary file 1 [file cancers-12-01213-s001.zip › cancers-790057 supplementary/Figure S4.pdf]
